# Nesting Habitat Suitability of the Kentish Plover in the Arid Lands of Xinjiang, China

**DOI:** 10.3390/ani13213369

**Published:** 2023-10-30

**Authors:** Peng Ding, Zitan Song, Yang Liu, Naerhulan Halimubieke, Tamás Székely, Lei Shi

**Affiliations:** 1 College of Animal Sciences, Xinjiang Agricultural University, Urumqi 830052, China; dpengde@126.com; 2 Xinjiang Key Laboratory for Ecological Adaptation and Evolution of Extreme Environment Biology, College of Life Sciences, Xinjiang Agricultural University, Urumqi 830052, China; 3 State Key Laboratory of Biocontrol, School of Ecology, Sun Yat-sen University, Shenzhen 518107, China; 13624326602@163.com (Z.S.); liuy353@mail.sysu.edu.cn (Y.L.); 4 Comparative Socioecology Group, Max Planck Institute of Animal Behavior, 78467 Konstanz, Germany; 5 Milner Centre for Evolution, Department of Biology and Biochemistry, University of Bath, Bath BA1 7AY, UK; n.halimubieke@ucl.ac.uk (N.H.); bssts@bath.ac.uk (T.S.); 6 Department of Evolutionary Zoology and Human Biology, University of Debrecen, H-4032 Debrecen, Hungary

**Keywords:** *Charadrius alexandrinus*, nesting location, habitat assessment, MaxEnt model, protection status

## Abstract

**Simple Summary:**

Wetlands in arid regions are crucial habitats for shorebirds, including the Kentish plover (*Charadrius alexandrinus*), and they are also heavily influenced by water resource management. This shorebird is very sensitive to its nesting habitat and serves as a good indicator for monitoring environmental changes in wetland ecosystems. Therefore, nesting habitat studies of the Kentish plover can reveal their survival status, habitat environment, and the threats they face, which are crucial for shorebird population recovery and habitat conservation in arid lands. The results of this study show that the primary nesting habitats of the plovers require multiple land use types near water bodies with little or no vegetation in order to cool their eggs and themselves with wet feathers, detect predators early, and adopt anti-predation strategies. It was also found that the proportion of suitable nesting habitat protected for Kentish plovers in the study area was low; therefore, regional wildlife and habitat protection is urgently needed in the study area. These findings can provide a reference for scientists to formulate practical habitat conservation and management measures for shorebirds in arid lands.

**Abstract:**

Understanding the main ecological factors of the nesting habitat of shorebirds is of great significance in relation to their protection and habitat management. Habitat loss and change due to a lack of water threaten the biodiversity of shorebirds, with impacts likely to be most pronounced in arid lands. We collected the data of 144 nesting sites and 10 ecological factors during the breeding season from April to July each year in 2019 and 2020 in nine river districts in Xinjiang. The MaxEnt model was applied to assess the suitability of nesting habitats for Kentish plovers (*Charadrius alexandrinus*) in the study area to examine the main factors affecting their nesting habitat. The most suitable nesting habitats are mostly distributed in plain reservoirs in the middle part of the Northern Slope of the Tianshan Mountains, Ebinur Lake and its eastern position in the southwestern Junggar Basin, near Ulungur Lake of the Ulungur river area and the southern Irtysh river area. The distance from water, normalized difference vegetation index, mean temperature of the breeding season, slope, and land use were the main factors affecting the nesting habitat selection of Kentish plovers. It was found that the proportion of suitable nesting habitat protected for the Kentish plovers in the study area was low (851.66 km^2^), accounting for only 11.02% of the total suitable nesting habitat area. In view of the scarcity and importance of water bodies in arid lands and the lack of protection for Kentish plovers at present, it is suggested to strengthen the conservation and management of the regional shorebirds and their habitats by regulating and optimizing the allocation of water resources.

## 1. Introduction

A habitat includes all of the biotic and abiotic resources needed by an organism for its survival and reproduction [1,2]. A suitable habitat can facilitate the continued existence of a species, and breeding habitats, in particular, are essential for ensuring population reproduction and recovery [3,4,5]. The scientific management of breeding habitats is crucial for species conservation [6,7,8]. Nesting is a very important stage in the life history of birds, and many studies of avian nesting success have examined nesting habitat characteristics and quality [9,10]. Therefore, accurately predicting the potential distribution of nesting habitats for a species and identifying key ecological factors are of great significance for species conservation and habitat management [11,12,13].

The habitat of birds is affected by many ecological factors, such as land use type, climate change, terrain conditions and human disturbance intensity [14,15,16]. Shorebirds are very sensitive to habitat quality, and they are an important part of the wetland ecosystem. They also play a critical role in maintaining the stability of wetland ecosystems and serve as good indicators for monitoring environmental changes in wetland ecosystems [17,18,19,20]. Many studies on the habitat of shorebirds have mainly focused on nest site selection, habitat utilization, and nest survival rate [14,15,16,18,20,21,22,23], although several studies have tested suitable breeding habitats via distribution modeling [11,24]. In recent years, with the gradual maturity of 3S technology, relevant model methods of species habitat research have made great progress. However, few studies have modeled the current breeding distributions of shorebirds using maximum entropy modeling [8,25]. Assessing habitat suitability and understanding the relative importance of ecological factors for shorebirds can provide valuable information for species conservation and management [21,26,27].

Northern Xinjiang has relatively abundant water resources in its arid land, providing ample food and suitable habitats for waterbirds such as shorebirds along global migratory routes, including Central Asia, East Africa, and West Asia [18,28,29]. It is also an important breeding habitat for birds in arid regions of China; therefore, the Ebinur Lake Wetland National Nature Reserve and Ulungur Lake National Wetland Park have been established in the area [30,31]. The wetlands surrounding natural inland lakes and plain reservoirs in arid regions are crucial habitats for waterbirds, including the Kentish plover (*Charadrius alexandrinus*) (hereafter referred to as the plover), and they are also heavily influenced by water resource management [32]. Understanding how breeding birds utilize these nesting habitats is a critical first step in predicting the habitat changes of these species [8]. Therefore, assessing the potential suitable nesting habitat zones for the plover is of great significance for the reproduction of shorebirds and biodiversity conservation.

In this study, we used the plover as an ecological model species [24,33] to investigate the impact of different ecological factors on nesting habitats in arid land. The following objectives were addressed: (1) to assess and predict the nesting habitats of the plover in Northern Xinjiang; (2) to explore the key ecological factors influencing the nesting habitats of the plover; (3) to analyze the conservation status of nesting habitats in Northern Xinjiang, aiming to provide recommendations for the conservation and habitat management of shorebirds and other waterbirds.

## 2. Materials and Methods

### 2.1. Study Area

**Figure 1:**
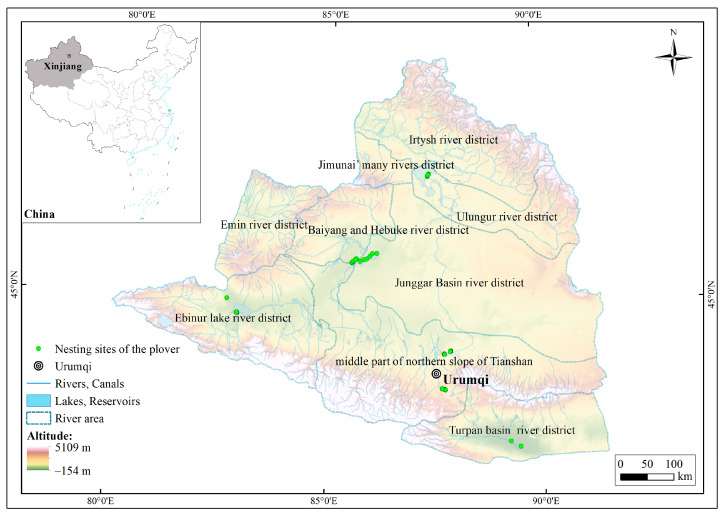
Diagram of the study area.

The research area included nine river districts located within the coordinate range of 79°51′ to 91°30′ E and 42°20′ to 49°11′ N (Figure 1); the total area of the study area was 377,307.88 km^2^ [34]. The elevation in the northern and southern parts of the research area was relatively high, with the Tianshan and Altai mountains reaching approximately 5200 m. The central part consisted of the lower-elevation Junggar basin, with the lowest point of Turpan basin being 154 m below sea level. The research area had a typical temperate continental arid and semi-arid climate, with an annual average temperature ranging from −4 to 9 °C. The summers are hot, and the winters are cold. The annual precipitation ranges from 150 to 200 mm [35]. Influenced by the westerlies, the research area received abundant snowfall, and the melting snow serves as the main water source, significantly impacting the surface water and wetland ecosystems in the research area [36,37].

### 2.2. Data Sources and Processing

#### 2.2.1. Nesting Site Data

The data on the plover nesting sites used in this study were obtained through direct observations from 2019 to 2020 during the breeding season in wetlands including Ebinur Lake, Manas Lake, Little Ailik Lake, Ulungur Lakem and Aydingkol Lake, as well as artificial plain reservoirs including Liuchengzi Reservoir and Wushihua Reservoir. These areas represent six river basins in Northern Xinjiang. During the survey, fixed-point observations were conducted using 20–60× monocular telescopes or 10× binocular telescopes [32]. When a plover was observed, the latitude and longitude information of the nesting site was recorded. A total of 144 plover nesting sites were recorded (Figure 1). As the spatial resolution of the ecological factors used in this study was 30 m, redundant data of nesting sites within the same ecological factor grid were removed using the ENMTools 1.4.4 software to avoid sampling bias. Finally, 132 valid plover nesting sites were selected for model prediction.

#### 2.2.2. Selection of Ecological Factors and Data Sources

The selected ecological factors related to the geographic distribution of the plovers, including climate factors, terrain factors, human activity factors, and vegetation factors. For modeling, we used climatic data collected at meteorological stations in the study area from the National Meteorological Information Center of the China Meteorological Administration during the breeding season from April to July in 2019 and 2020 [38]. These data were interpolated into 30 m resolution raster images using the kriging function in ArcMap 10.4. The terrain, road, and settlement data were sourced from the Resource and Environment Science Data Center of the Chinese Academy of Sciences [39]. The slope and aspect were extracted from the elevation data using ArcGIS 10.4 software. The distances to roads and settlements were calculated using spatial analysis tools in ArcGIS 10.4 based on road and settlement vector data to obtain Euclidean distances. The spatial resolution for both the terrain and disturbance factors was 30 m. The land use data, among the other factors, were obtained from the 2020 global 30 m surface cover data (GlobeLand30) from the National Geomatics Center of China [40], with a spatial resolution of 30 m. The distance to water bodies, among the other factors, was calculated using spatial analysis tools in ArcGIS 10.4 based on the water body data within the land use data, resulting in Euclidean distances. The normalized difference vegetation index data were derived from Landsat 8 remote sensing images, which were radiometrically calibrated and atmospherically corrected [39]. A maximum value composition and mosaic were performed, with a spatial resolution of 30 m. The data on the nature reserves were obtained from the CAS Earth Big Earth Data Science Project—Biodiversity and Ecological Security Big Data Integration Platform [41]. There were 79 natural protected areas of various types in the study area, including 17 nature reserves and 20 wetland parks. The cumulative protected area of the aforementioned reserves was approximately 45,196.43 km^2^, accounting for 11.98% of the total area of Northern Xinjiang [30,31].

**Figure 2:**
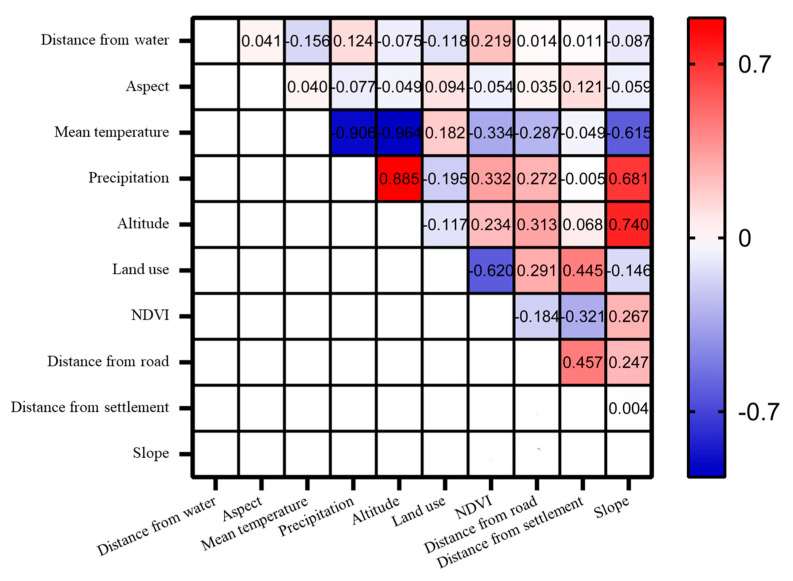
Correlation matrix of the ecological factors on a geographic scale in the study area. The darker the blue and red squares, the greater the correlation between the two ecological factors.

The ecological factors used in this study included climatic, human interference, terrain, and vegetation factors (Table 1). According to the requirements for data consistency analysis using the MaxEnt 3.4.4 model, all the ecological factor data should be clipped based on the size of the study area in ArcGIS 10.4 software. The spatial resolution should be uniformly resampled to 30 m, ensuring consistency in the boundaries, coordinate systems, raster sizes, row, and column numbers of all ecological factors [42]. A Spearman rank correlation analysis was performed to examine the correlation between different climate factors [43]. Ecological factors with correlation coefficients greater than 0.70 or less than −0.70 were deemed to be of significant indicative value (Figure 2). The higher percentage contribution and permutation importance of ecological factors were retained, and the altitude and precipitation factors were removed. Finally, the remaining eight selected ecological factors were converted to an ASCII format for model computation.

### 2.3. Model Operation and Effect Evaluation

#### 2.3.1. Model Building

We utilized a maximum entropy model (MaxEnt) to evaluate the nesting habitat suitability of the plovers based on the nesting sites and ecological factor data in the MaxEnt 3.4.3 software. We randomly selected 75% of the nesting site data as the training set and the remaining 25% as the testing set. The model was repeatedly run 10 times. Using the Jackknife method, we calculated the percent contribution of each ecological factor to the model predictions. The software can infer the main ecological factors that restrict the nesting habitat of the plovers and plot the response curves of each ecological factor. Default values were used for the remaining parameters. The results were output in a Logistic format, and the average of 10 repeated simulations was selected as the final simulated result. Finally, we generated a suitability map for the nesting habitat of the plovers.

#### 2.3.2. Verification of the Model

We used the receiver operating characteristic (ROC) curve analysis method to predict the nesting habitat suitability for the plovers. The sensitivity and specificity were predicted, and the area under the ROC curve (AUC) was obtained as an indicator of the model’s predictive accuracy [44,45]. The accuracy of the model prediction is directly proportional to the AUC value, which ranges from 0 to 1 [46,47]. A higher AUC value indicates a more accurate prediction. Generally, AUC values between 0.50 and 0.60 indicate model prediction failure, between 0.61 and 0.70 indicate a poor performance, between 0.71 and 0.80 indicate a fair performance, between 0.81 and 0.90 indicate a good performance, and between 0.91 and 1 indicate an excellent performance [13].

#### 2.3.3. Assessment of Importance

The impact of different ecological factors on the suitability of nesting habitats for the plovers was determined through an analysis of the composite cutting method (Jackknife test), percent contribution, and permutation importance [48,49]. These analyses assess the degree of influence of ecological factors and identify the main factors affecting the nesting habitat of the plovers. Factor response curves were then plotted to analyze the impact patterns of each ecological factor on the nesting habitat suitability for the plovers.

#### 2.3.4. Classification of Suitability

We imported the average ASC file generated from the MaxEnt analysis into ArcGIS 10.4. First, we used the “ASCII to Raster” tool in ArcGIS 10.4 to convert the results into a raster layer and perform the necessary projection. Then, we proceeded with visual editing. Next, we utilized the reclassification tool in the spatial analysis toolbox of ArcGIS 10.4 to classify the suitability of the breeding habitats for the plovers. We employed the natural breaks classification method and the maximum training sensitivity plus specificity threshold to determine the thresholds and divided them into three categories [45,50,51]: most suitable nesting habitat (*p* ≥ 0.4), second suitable nesting habitat (0.1 ≤ *p* < 0.4), and unsuitable nesting habitat (*p* < 0.1). Finally, we imported the converted model predictions into ArcGIS 10.4 software to create a distribution map and calculate the area of suitable nesting habitats at different levels for the plovers.

## 3. Results

### 3.1. Model Performance

**Figure 3:**
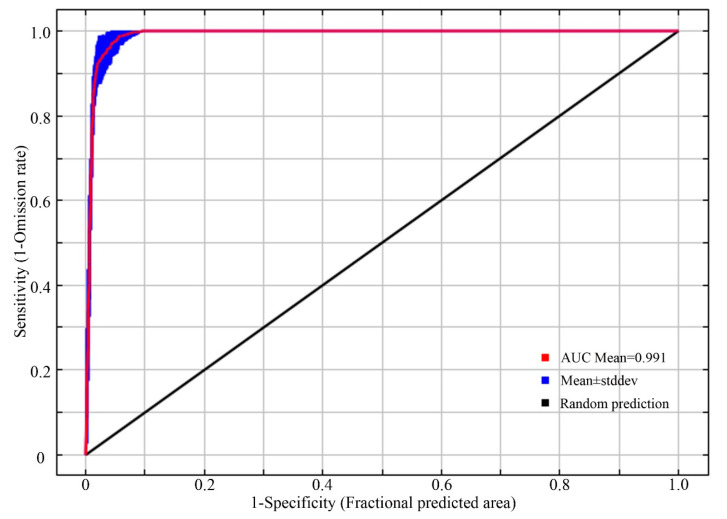
ROC curve for verification.

The ROC curve evaluation results based on the MaxEnt model, repeated 10 times, are shown in Figure 3. The AUC value of the model on the test set from 2020 was 0.991 ± 0.003, with all the AUC values ranging from 0.9 to 1. This indicates that the accuracy of the model’s predictions in this study reached a “high” level. The model’s predictions are reasonable and reliable, making them suitable for simulating the distribution of suitable nesting habitats for the plovers in the northern region of Xinjiang and studying their relationship with ecological factors.

### 3.2. Importance of the Ecological Factors

The selection of ecological factors is crucial in determining the accuracy of the model; therefore, it is necessary to evaluate which ecological factors contribute the most to the model. According to the contribution rates and permutation importance of each ecological factor, as shown in Table 2, in the model prediction, the distance from water, NDVI, mean temperature, slope, and land use contributed significantly. Their cumulative contribution rate reached 96.80%, which was significantly higher than the other factors. Comparing the permutation importance, it was found that the distance from water, NDVI, and mean temperature had high permutation importance and played a crucial role in the modeling process.

**Figure 4:**
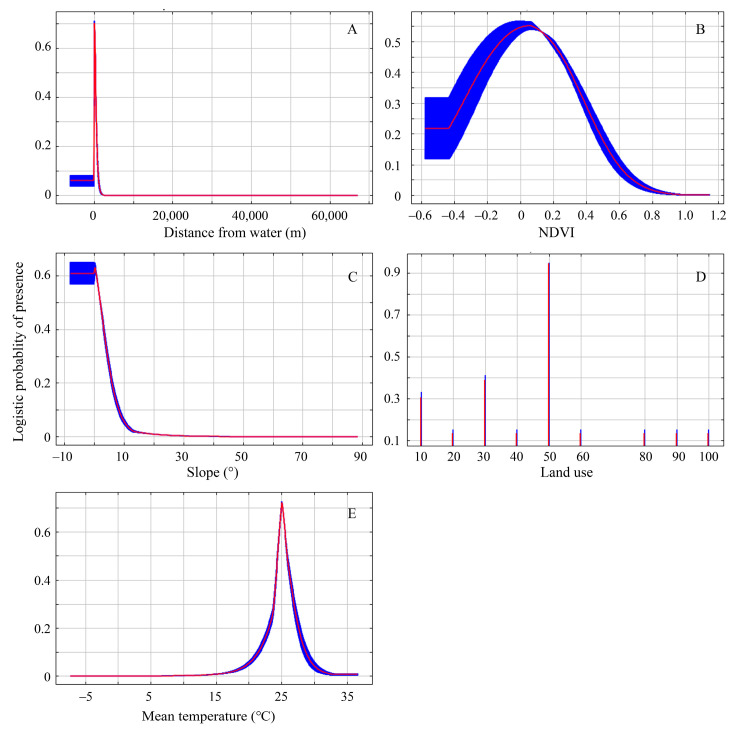
Response curves of the major ecological factors for model prediction. (**A**–**E**) Distance from water, NDVI, slope, land use, mean temperature. The red curve and blue shading indicate the mean and the mean ± one standard deviation of the logistic probability of presence for the 10 MaxEnt replicate runs, respectively. The abscissa value of Figure D to represents the following land use types: 10—cultivated land, 20—forest, 30—grassland, 40—shrubland, 50—wetland, 60—water bodies, 70—tundra, 80—artificial surfaces, 90—bare land, 100—permanent snow and ice.

According to the Jackknife test analysis, the distance from water, NDVI, mean temperature, slope, and land use were the primary ecological factors that had the greatest impact on the nesting habitat suitability of the plovers. The response curves of each ecological factor in predicting the distribution of the plovers can reflect the relationship between different distribution probabilities and the values of the main ecological factors (Figure 4).

### 3.3. Nesting Habitat Suitability Maps for C. alexandrinus

**Figure 5:**
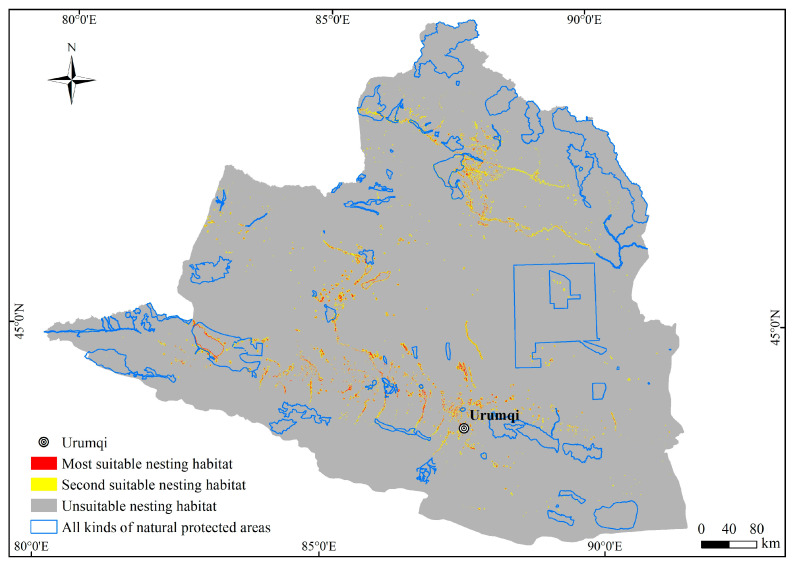
Distribution of suitable nesting habitat of the plovers in Northern Xinjiang.

The primary nesting habitats of the plovers are distributed in four river regions in northern Xinjiang, namely the plain reservoir in the middle section of the northern slope of the Tianshan Mountains, the Ebinur Lake in the southwestern part of the Junggar Basin and its eastern region, the vicinity of the Ulungur Lake in the Ulungur River region, and the southern part of the Irtysh River region (Figure 5). The total area of suitable nesting habitats for the plovers in Northern Xinjiang is 7727.32 km^2^, of which the most suitable nesting habitat covers an area of 1913.71 km^2^, accounting for 24.77% of the nesting habitat area. The second suitable nesting habitat covers an area of 5813.61 km^2^, accounting for 75.23% of the nesting habitat area.

### 3.4. Conservation Status of the Plover Nesting Site

By overlaying the predicted suitable nesting habitats of the plovers with the natural protected areas in Northern Xinjiang, it was determined that there were 851.66 km^2^ of suitable nesting habitats for the plovers within the protected areas, accounting for only 11.02% of the total suitable nesting habitat area (Table 3).

## 4. Discussion

### 4.1. Major Ecological Factors Influencing Habitat Assessment

Our study found that the distance from water where the nests of the plovers were located was particularly important in arid land. Like other populations around the world, the plovers nest in a variety of land use types, such as wetlands, desert grasslands, and derelict lands, taking into account food, water, and shelter [17,20,24,32,33].

Firstly, the distance from water was the most significant factor influencing the distribution of nesting habitats for the plovers, and it contributed the highest proportion (77.30%) to the suitability of the nesting habitats for the plovers. The nests were located at a certain distance from the water to avoid the inundation of nests during the incubation stage, but not too far to make sure the chicks would not have to expend too much energy getting to the water. As a precocious bird, soon after the birds emerge from their shells, the chicks are capable of independent walking and foraging, and they subsequently move towards the vicinity of water [15,52]. This is primarily due to the fact that plovers and their chicks feed near water bodies, where food abundance is higher, facilitating rapid energy replenishment for the birds [22,23,53]. At the same time, faced with higher ambient temperatures in arid land during the breeding season, parents need to fly to the water’s edge and wet their ventral plumage so that they can cool their eggs and themselves [54,55]; therefore, their nests will not be built in an area far from water.

Secondly, the normalized difference vegetation index and slope were also important factors influencing the distribution of nesting habitats for the plovers. This may have something to do with the plovers’ anti-predation strategy. Studies have shown that the species breeding in high mountain and Arctic tundra regions are becoming increasingly vulnerable to predation with increasing vegetation cover and primary productivity due to climate change [6,11,56]. Our results suggest that the plovers of arid land often select flat areas with little to no vegetation around the normalized difference vegetation index of approximately 0.1. This facilitates the use of the predator detection anti-predation strategies to reduce the risk of predation [57,58].

Thirdly, the plovers exhibit a significant preference for habitats consisting of wetlands, grasslands, and bare lands near water bodies. Among the major ecological factors, in terms of land use types, these habitats are more likely to occur in wetlands. Therefore, there is strong evidence indicating the vital importance of wetlands, grasslands, and bare lands near water bodies for the breeding of the plovers in the arid land of Northern Xinjiang.

### 4.2. The Plasticity of the Nest to Land Use

Extensive research has been conducted on the nesting habitat and nest site selection of the plover [15,21,23,33,56,59,60]; however, less focus has been placed on the nesting habitats of the plovers in the arid lands of Xinjiang, China. This study discovered that these plovers choose a variety of land use types at suitable distances from water in arid lands, such as wetlands, grasslands, farmlands, and so on (Figure 4). This was consistent with the various nesting habitats of the plovers, including natural habitats such as alkaline grasslands, marshes, saline lakes, shallow water coastal lagoons, sandy beaches, and rocky shores, as well as anthropogenic habitats, like agricultural fields and salt pans in other areas of the globe [15,23,59,60,61,62,63,64]. The nesting sites of the plover in Northern Xinjiang conform to the aforementioned adaptable land use types, primarily concentrated in natural habitats such as wetlands, grasslands, and bare ground near water bodies. In recent years, some natural habitats near water bodies in Northern Xinjiang have been converted into farmland. However, this study discovered that the plovers also nest in human-modified habitats such as abandoned fields close to water bodies with no vegetation. Research suggests that in coastal areas where natural habitats have been lost or degraded, certain artificial habitats such as salt pans can serve as alternative or supplementary nesting sites for plovers and other waterbirds under certain conditions [65,66,67,68]. Due to the lack of systematic monitoring of breeding populations of plovers and other waterbirds in Northern Xinjiang in the past, it has been challenging to establish a link between nesting habitats and the aforementioned land use changes. During the two-year investigation conducted in this study, plover nesting in Northern Xinjiang exhibited significant flexibility. For example, in June 2019, after artificial regulation of the water levels in the Wushihua Reservoir, a second peak of egg-laying occurred as the suitable breeding area expanded [23]. The plovers showed adaptability by selecting specific land use types for nesting based on factors such as distance from water, NDVI, and slope.

### 4.3. Conservation Gaps in the Nesting Habitat of C. alexandrinus

A favorable nesting habitat is a prerequisite for the reproduction and population recovery of species. Proper planning and establishment of wetland nature reserves are effective approaches to protect plovers and sandpipers [69,70,71]. According to Table 3, currently, 851.66 km^2^ of suitable nesting habitat is protected within the aforementioned nature reserves, accounting for only 11.02% of the area. Among them, suitable nesting habitats for the plovers in the Ebinur Lake, Ulungur River, and Irtysh River are largely protected by various wetland nature reserves and wetland parks. However, the large area of suitable nesting habitat for the plovers in the middle section of the northern slope of the Tianshan Mountains needs to improve its level of protection. Additionally, the water bodies in the arid lands of Northern Xinjiang originate from glaciers and permanent snow in mountainous areas [72]. Climate change can directly or indirectly affect regional water bodies and the wetland habitats near them [8,15,22,73,74]. Therefore, predicting the potential nesting habitats of the plovers under different climate scenarios in the future is of significant importance for understanding the species diversity and ecosystem stability of wetlands in arid lands under global climate change, and it can be considered as a direction for future research.

## 5. Conclusions

The identification of the ecological factors affecting the nesting habitat preferences of species is key to understanding the relationships between habitat features and species’ distributions. The results of this study indicated that the distance from water, normalized difference vegetation index, mean temperature, slope, and land use were the major ecological factors influencing the nesting habitat distribution of the plovers. There was 851.66 km^2^ of suitable nesting habitat for the plovers within the protected areas of the research area, accounting for only 11.02% of the total suitable nesting habitat area. In the future, it is recommended to focus on the nesting habitat of the plovers in plain reservoirs within the middle section of the northern slope of the Tianshan Mountains, strengthen conservation management in conjunction with practical measures, increase the coverage of nature reserves, and better protect the waterbirds and their habitats in the arid lands of Xinjiang.

## Figures and Tables

**Table 1:** Descriptions of the ecological factors in the model.

Ecological Factors	Description, Unit	Data Source
Mean temperature	Mean temperature during the breeding season from April to July, °C	[38]
Precipitation	Precipitation during the breeding season from April to July, mm	[38]
Altitude	Vertical elevation of the ground above or below sea level, m	[39]
Slope	Degree of steepness of a surface unit, °	[39]
Aspect	Direction of the projection of a slope normal onto a horizontal plane, °	[39]
Distance from road	Distance to nearest road, m	[39]
Distance from settlement	Distance to nearest settlement, m	[39]
Distance from water	Distance to nearest water, m	[40]
Land use	Land use type (cultivated land, forest, grassland, shrubland, wetland, water bodies, tundra, artificial surfaces, bare land, permanent snow and ice)	[40]
NDVI	Normalized difference vegetation index	[39]

**Table 2:** Percent contribution and Permutation importance of ecological factors.

Factors	Percent Contribution (%)	Permutation Importance (%)
Distance from water	77.3	86.2
NDVI	8.4	7.4
Mean temperature	6.2	3.6
Slope	2.1	0.9
Land use	3.1	0.4
Distance from settlement	1.8	0.9
Aspect	0.8	0.1
Distance from road	0.3	0.5

**Table 3:** Conservation status of suitable nesting habitats for the plovers in Northern Xinjiang.

Area	Area and Proportion	Protected	Unprotected	Total
Northern Xinjiang	Area (km^2^)	45,196.43	332,111.45	377,307.88
	Proportion (%)	11.98	88.02	100.00
Most suitable nesting habitat	Area (km^2^)	245.94	1667.77	1913.71
	Proportion (%)	12.85	87.15	100.00
Second suitable nesting habitat	Area (km^2^)	605.72	5207.89	5813.61
	Proportion (%)	10.42	89.58	100.00

## Data Availability

The data presented in this study are available from the corresponding author upon request.
